# Effectiveness of a brief intervention for alcohol consumption among Brazilian women in a household setting

**DOI:** 10.1186/1940-0640-8-S1-A31

**Published:** 2013-09-04

**Authors:** Carla FP Gebara, Fernanda MC Bhona, Marcel T Vieira, Cleusa P Ferri, Lelio M Lourenço, Ana R Noto

**Affiliations:** 1 Department of Psychobiology, Universidade Federal de São Paulo, São Paulo, Brazil; 2 Department of Psychology, Universidade Federal de Juiz de Fora, Juiz de Fora, Brazil; 3 Department of Statistics, Universidade Federal de Juiz de Fora, Juiz de Fora, Brazil

## 

At-risk alcohol use is increasing among women and it has been linked to important harm to their health. However, the effectiveness of brief intervention (BI) among women is unclear. Research on the most effective components of those interventions in the female population is necessary, especially in low and middle income countries. The aim of this study was to evaluate the impact of a BI, carried out among women in a household community setting, on their alcohol consumption. Participants were enrolled through a household survey, using a probabilistic sample of 905 adult women in the city of Juiz de Fora, Brazil. The 61 (6.7%) women who screened positive for at-risk drinking (AUDIT score ≥ 8) were randomly allocated to one of two groups: the Intervention Group (n=32), which received one single session of BI carried out in the household setting after screening, or the Control Group (n=29) which did not receive BI. After three months, 46 (75.4%) participants completed the follow-up interview (23 in each group). All participants received an informative leaflet, and the Intervention Group received a 15-40 minute BI, which provided information on the adverse consequences of alcohol and strategies to help moderate consumption. Participants of both groups decreased their alcohol consumption after three months. The intervention group decreased the mean AUDIT score from 12.4 to 9.4, while the control group reduced from 10.6 to 7.5. Preliminary analyzes using general linear modelling indicate that there was no difference between the Intervention Group and the Control Group in relation to the main outcome (total AUDIT score). Although no difference was found between groups, the results of this study are not conclusive and may reflect the small sample size of the trial. More studies are needed to better estimate the effectiveness of BI among women in a household setting.

